# Maternal Perceptions and Practices in Trachoma Prevention: A Descriptive and Correlational Analysis Using the Health Belief Model in Rural Ethiopia

**DOI:** 10.1002/hsr2.72401

**Published:** 2026-04-16

**Authors:** Zufan Alamrie Asmare, Almaw Genet Yeshiwas, Gashaw Melkie Bayeh, Tilahun Degu Tsega, Sintayehu Simie Tsega, Asaye Alamneh Gebeyehu, Getaneh Atikilt Yemata, Rahel Mulatie Anteneh, Amare Genetu Ejigu, Ahmed Fentaw Ahmed, Zeamanuel Anteneh Yigzaw, Abathun Temesgen, Abebaw Molla Kebede, Habitamu Mekonen, Anley Shiferaw Enawgaw, Getasew Yirdaw, Berhanu Abebaw Mekonnen, Meron Asmamaw Alemayehu, Zekaryas Ewnetu Gashu, Chalachew Yenew

**Affiliations:** ^1^ Department of Ophthalmology, School of Medicine and Health Science Debre Tabor University Debre Tabor Ethiopia; ^2^ Department of Environmental Health, College of Medicine and Health Science Injibara University Injibara Ethiopia; ^3^ Department of Public Health College of Medicine and Health Sciences, Injibara University Injibara Ethiopia; ^4^ Department of Epidemiology School of Public Health, Cheeloo College of Medicine, Shandong University Jinan China; ^5^ Department of Medical Nursing, School of Nursing, College of Medicine and Health Science University of Gondar Gondar Ethiopia; ^6^ Depatment of Public Health, College of Health Science Debre Tabor University Debre Tabor Ethiopia; ^7^ Department of Midwifery College of Medicine and Health Sciences, Injibara University Injibara Ethiopia; ^8^ Facility of Health Science and Technology, University of Brazilia Brasília Brazil; ^9^ Department of Health Promotion and Behavioral Sciences School of Public Health, College of Medicine and Health Sciences, Bahir Dar University Bahir Dar Ethiopia; ^10^ Department of Human Nutrition College of Health Science, Debre Markos University Debre Markos Ethiopia; ^11^ Department of Public Health College of Health Sciences, Debre Markos University Debre Markos Ethiopia; ^12^ Department of Environmental Health Science College of Medicine and Health Sciences, Debre Markos University Debre Markos Ethiopia; ^13^ Department of Nutrition and Dietetics School of Public Health, College of Medicine and Health Sciences, Bahir Dar University Bahir Dar Ethiopia; ^14^ Department of Epidemiology and Biostatistics Institute of Public Health, College of Medicine and Health Sciences, University of Gondar Gondar Ethiopia; ^15^ Department of Pediatrics and Child Health, College of Medicine and Health Sciences University of Gondar Gondar Ethiopia; ^16^ Department of Public Health Debre Tabor University Debra Tabor Ethiopia; ^17^ Jockey Club College of Veterinary Medicine and Life Sciences City University of Hong Kong Hong Kong China

**Keywords:** correlation analysis, health belief model, health knowledge, health perception, maternal health, neglected tropical diseases, rural Ethiopia, trachoma

## Abstract

**Background and Aims:**

Trachoma remains a leading cause of preventable blindness in Ethiopia, where maternal preventive practices are critical for interrupting transmission. However, the psychosocial and perceptual landscape shaping these practices in high‐burden rural communities is poorly characterized. This study describes maternal health beliefs and examines their correlational structure using the Health Belief Model (HBM) to inform the content and delivery of health education.

**Methods:**

A community‐based cross‐sectional study was conducted in June 2022 in the Andabet district, Northwest Ethiopia, involving 624 mothers with children under nine. Data was collected via face‐to‐face interviews using a validated HBM‐based questionnaire. The analysis included descriptive statistics of socio‐demographics and HBM constructs, as well as a Pearson correlation matrix to explore the relationships between perceived susceptibility, severity, benefits, barriers, self‐efficacy, and prevention practices.

**Results:**

Nearly half (49.5%) of mothers reported good trachoma prevention practices. Descriptive analysis revealed a high perceived severity (mean = 0.97, SD = 0.02) and strong belief in benefits (mean = 0.85, SD = 0.06), but moderate perceived susceptibility (mean = 0.65, SD = 0.20) and self‐efficacy (mean = 0.68, SD = 0.21). A critical knowledge gap was identified, with only 38.5% recognizing flies as a transmission vector. Correlation analysis revealed negligible linear relationships between individual HBM constructs and prevention practices (all |*r*| < 0.1), as well as weak inter‐construct correlations, suggesting independent, non‐linear influences on behavior.

**Conclusion:**

While mothers recognize trachoma's severity and value prevention, specific knowledge gaps and moderate self‐efficacy levels persist. The lack of strong linear correlations highlights the complexity of health behavior, indicating that improving practices requires more than simply raising awareness. Health education must be multi‐faceted, targeted, and designed to address specific misconceptions (e.g., fly transmission) while bolstering personal confidence and competence in performing preventive actions within resource‐constrained realities.

## 1 Background

Trachoma, a neglected tropical disease caused by *Chlamydia trachomatis*, is hyperendemic in rural Ethiopia, where poor sanitation and limited access to water perpetuate its transmission. Trachoma remains a leading cause of preventable blindness globally, with the burden disproportionately affecting low‐resource settings, including rural Ethiopia [1]. The World Health Organization's SAFE strategy (Surgery, Antibiotics, Facial cleanliness, and Environmental improvement) is the cornerstone of trachoma prevention. Among these, promoting facial cleanliness and environmental hygiene is particularly critical in breaking the transmission cycle. Mothers, as primary caregivers, play a pivotal role in ensuring children adopt these preventive practices [2].

While behavioral interventions are key to reducing trachoma transmission, understanding the psychosocial and cultural factors influencing maternal adoption of preventive measures remains limited. Existing studies have primarily focused on clinical or environmental interventions, with insufficient exploration of behavioral determinants such as perceived susceptibility, severity, benefits, and barriers as described in the Health Belief Model (HBM) [3]. Exploring maternal behaviors through the lens of the HBM offers a structured approach to identifying cognitive and emotional factors that influence health‐related behaviors. Addressing these gaps can inform the design of targeted, culturally appropriate interventions that empower mothers to prioritize and sustain trachoma prevention measures in their households [4].

The World Health Organization's SAFE strategy emphasizes the “F” and “E” components, which rely heavily on sustained community behavior change. Mothers, as primary caregivers, play a pivotal role in adopting and promoting these practices within households. While prior research has documented clinical prevalence and some knowledge gaps, a detailed, theory‐based mapping of maternal perceptions is lacking. The HBM provides a robust framework for systematically evaluating the cognitive and perceptual factors (perceived susceptibility, severity, benefits, barriers, and self‐efficacy) that precede health‐related action [5]. Understanding the descriptive profile and interrelationships of these constructs in a specific cultural context is essential for moving beyond generic awareness campaigns to design nuanced, resonant health messages. This study aims to fill this gap by providing a comprehensive descriptive and correlational analysis of HBM constructs among mothers in a trachoma‐endemic district of Ethiopia, thereby establishing a foundational evidence base for targeted social and behavior change communication (SBCC) interventions.

## 2 Methods

### 2.1 Study Design, Setting, and Participants

This community‐based cross‐sectional study was conducted in June 2022 in the Andabet district, South Gondar Zone, Northwest Ethiopia. Andabet is a rural, trachoma‐hyperendemic district with a 2017 Trachomatous Follicular (TF) prevalence of 37% among children. The study included 634 mothers aged 15 years and above, each with at least one child under the age of nine, who were permanent residents of their kebele (the smallest administrative unit) for a minimum of 6 months. Participants were selected using a multistage random sampling technique. First, six out of the district's 26 *kebeles* were randomly selected. The sample size was proportionally allocated to each selected *kebele* based on the number of eligible mothers. Households were then chosen via systematic random sampling from comprehensive lists, and one eligible mother per household was interviewed. This sampling methodology is a component of a larger survey. This analysis specifically concentrates on the subset of data related to the HBM and trachoma prevention practices. The report complies with STROBE guidelines to ensure high‐quality reporting [6].

### 2.2 Data Collection Instrument and Procedures

Data was collected through face‐to‐face interviews using a structured, pre‐tested questionnaire. The instrument was developed based on the HBM framework and comprised four main Section [7]:

1. **Socio‐demographic Characteristics:** This section captured information on age, residence, educational level, occupation, household income, family size, and access to water and sanitation facilities [8].
2. **Knowledge of Trachoma:** This section assessed knowledge regarding causes, modes of transmission, symptoms, and preventive measures of trachoma [8].
3. **Health Belief Model Constructs:** This core section measured the five HBM domains: **Perceived Susceptibility:** Beliefs about personal/family risk of contracting trachoma (5 items) [9], **Perceived Severity:** Beliefs about the seriousness of trachoma and its consequences (5 items), **Perceived Benefits:** Beliefs in the efficacy of recommended preventive actions (4 items), **Perceived Barriers:** Perceived obstacles to adopting preventive behaviors (4 items) and **Self‐Efficacy:** Confidence in one's ability to perform preventive behaviors (3 items) and finally, responses for HBM items were recorded on a binary (Yes/No) or a 5‐point Likert scale and later dichotomized for analysis [10].
4. **Trachoma Prevention Practices:** It was assessed by self‐reported adherence to key preventive behaviors, such as child facial cleanliness, proper waste disposal, and latrine use. Practices were classified as “good” or “poor” based on a composite score [8].

The questionnaire was originally prepared in English, translated into Amharic (the local language), and backtranslated to ensure conceptual consistency. Pre‐testing was conducted on 5% of the sample (32 mothers) in a non‐selected *kebele* to assess clarity, flow, and reliability. Cronbach's alpha for the HBM constructs ranged from 0.78 to 0.86, indicating good internal consistency. Ten trained data collectors (diploma‐level nurses fluent in Amharic) administered the interviews. A 2‐day training course covered the study objectives, ethical conduct, interview techniques, and details of the questionnaire. Supervisors monitored the fieldwork daily.

### 2.3 Data Quality Assurance

Multiple strategies were employed to ensure the quality of the data. The questionnaire was pre‐tested and refined. Regular field supervision and spot‐checking were conducted. Completed questionnaires were reviewed for completeness and consistency at the end of each day before being entered into the data.

### 2.4 Data Analysis

Data analysis was performed using R software (v4.5.1). Descriptive statistics (frequencies, percentages, means, and standard deviations) were used to summarize socio‐demographic characteristics, individual HBM responses, and composite scores for each HBM domain. Composite scores for each construct were calculated as the mean of its items and scaled from 0 to 1 for interpretation.

For correlational analysis, a Pearson's correlation matrix was generated to examine bivariate linear relationships between the continuous composite scores of the five HBM constructs and the binary prevention practice outcome. The strength and direction of correlations (r) were interpreted to understand the simple linear associations between maternal perceptions and reported behavior. This analysis was exploratory in nature, aiming to describe the pattern of relationships within the HBM framework in this specific context.

## 3 Results and Discussion

### 3.1 Socio‐Demographic Profile of Mothers With Children Under 9 Years in Andabet, Northwest Ethiopia

A total of 624 mothers participated in this study, achieving a response rate of 98.42%. The sociodemographic profile of these participants, detailed in Table 1, reveals distinct characteristics that are crucial for informing maternal and child health interventions. Each of these factors is supported by relevant scholarly context. The concentration of mothers (51%) in the 25–34‐year age bracket, with a further 34% aged 35 and above, indicates a mature cohort in their peak reproductive years, a demographic focal point for targeted maternal health services as established in reproductive health literature. The overwhelming majority reside in rural areas (72%), underscoring the imperative to design interventions for settings where geographic and systemic barriers to healthcare access are most acute, a well‐documented challenge in the Ethiopian context. Therefore, health policy must prioritize the development of targeted, community‐based intervention programs specifically designed for rural, agrarian populations with low formal education. These policies should mandate the use of visually based, low‐literacy communication strategies and deploy trained community health workers to deliver essential maternal and child health education directly within these hard‐to‐reach communities. A profound barrier identified is the low level of formal education, where 55% have no schooling, and community illiteracy is high (60.74%). This severely limits health literacy, necessitating the use of visual and oral communication strategies in any intervention program, a principle strongly supported by public health implementation science. The occupational profile, dominated by farming (51%) and domestic work (27%), reflects an economic reality where livelihood demands directly shape time, resources, and ability to seek healthcare, a key determinant of health access highlighted in socio‐economic studies. Therefore, transforming the demographic data on illiteracy from a passive characteristic into an active design principle, it is mandated that any intervention intended for this population must be architected from the ground up to bypass the written word and embed health knowledge through culturally resonant, practical, and accessible means (Table 1).

**Table 1:** Socio‐demographic characteristics of mothers having children aged under 9 years in Andabet, Northwest Ethiopia, 2022 (*n* = 624).

Characteristic	Category	*n* = 624	%
Mother's age	15–24	92	15
	25–34	321	51
	35 yrs and above	211	34
Residence	Rural	449	72
	Urban	175	28
Education	No formal education	341	55
	Primary	168	27
	Secondary & above	115	18
Occupation	Daily laborer	38	6
	Farmer	316	51
	Government employee	31	5
	Housewife	170	27
	Merchant	69	11
Received health education	Yes	71	11
	No	553	89

### 3.2 Trachoma Prevention Practices Among Mothers With Children Under 9 Years

**Figure 1:**
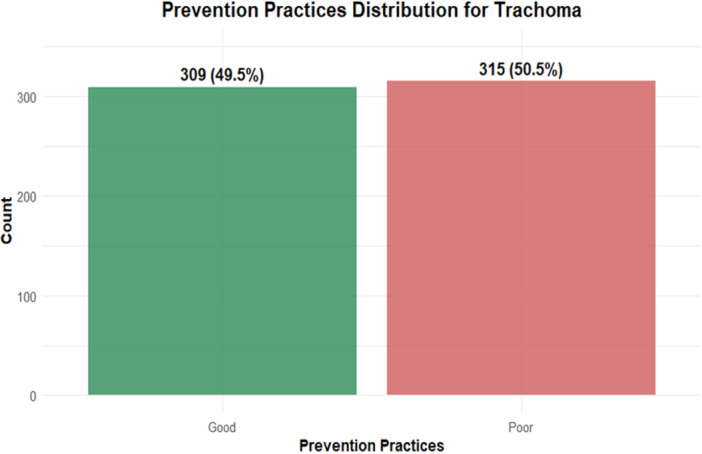
Trachoma prevention practices among mothers with children under 9 years in Andabet district, Northwest Ethiopia, 2022.

The bar graph above illustrates the distribution of good and poor trachoma prevention practices among mothers with children under 9 years old in Andabet District, Northwest Ethiopia. This visualization shows that there are 309 good practice cases (49.5%) and 315 poor practice cases (50.5%). The distribution is almost equal, with a very slight majority of poor prevention practices (50.5% vs. 49.5%). This suggests that prevention practices for trachoma in the studied population are nearly evenly split between good and poor, indicating a significant need for improvement in preventive measures (Figure 1).

### 3.3 Health Beliefs Status on Trachoma‐Prevention Perceptions Among Mothers of Young Children in Northwest Ethiopia

Based on the data presented in Table 2, the application of the HBM reveals a population with a strong foundational understanding of trachoma's severity and benefits of prevention, yet with specific, critical gaps in knowledge and practice that must be addressed by targeted interventions, The analysis of Perceived Susceptibility shows that most mothers correctly identify direct contact (69.9%) and family members (69.6%) as risk factors, and strongly associate the disease with farming (71.2%), reflecting an understanding grounded in their daily environment. Additionally, the idea that farm workers (71.2%) and unborn children (67.9%) are vulnerable to trachoma significantly influences perceptions. However, a major gap persists, as only 38.5% recognize flies as a key vector for transmission. This indicates that health education has successfully conveyed some routes of infection but has critically failed to embed knowledge of this primary environmental mechanism, leaving a significant loophole in preventive practices. Conversely, Perceived Severity is nearly universally acknowledged. An overwhelming majority (97.9%) understands the importance of hygiene, and 96.6% recognize the importance of face washing. Almost all (99.5%) recognize trachoma as a severe, potentially fatal (97.1%), and chronic (96.5%) disease. This near‐unanimous consensus on severity is a powerful asset, indicating the community is primed to accept and act on prevention messages, as they view the threat as serious and legitimate.

**Table 2:** Health belief model (HBM) constructs among mothers with children aged under 9 years in Andabet, Northwest Ethiopia, 2022 (*n* = 624).

HBM construct	Variable	Category	Frequency	Percent (%)
Perceived susceptibility	Direct contact with trachoma	No	178	28.5
		Yes	436	69.9
	Fly as a vector	No	374	59.9
		Yes	240	38.5
	Family members at risk of trachoma	No	180	28.8
		Yes	434	69.6
	Health of farmworkers hurt by trachoma	No	170	27.2
		Yes	444	71.2
	Health of unborn children hurt by trachoma	No	190	30.4
		Yes	424	67.9
Perceived severity	Hygiene practices for prevention of trachoma	No	3	0.5
		Yes	611	97.9
	Face washing	No	11	1.8
		Yes	603	96.6
	Trachoma severity perception	No	0	0.0
		Yes	621	99.5
	Chronic effects of trachoma	No	12	1.9
		Yes	602	96.5
	Fatality from trachoma	No	8	1.3
		Yes	606	97.1
Perceived benefits	Sanitation practices	No	245	39.3
		Yes	369	59.1
	Prevention of blindness in health education	No	6	1.0
		Yes	608	97.4
	Eye drop usage perceived as best treatment	No	0	0.0
		Yes	616	98.7
	Preventing spread through face washing	No	0	0.0
		Yes	614	98.4
		
Perceived barriers	Cultural beliefs: Trachoma as a disease of god	Agree	43	6.9
		Disagree	571	91.5
	Traditional medicine for healing trachoma	Agree	60	9.6
		Disagree	554	88.8
	High cost of SAFE strategy	Agree	50	8.0
		Disagree	564	90.4
	Social stigma of trachoma	Agree	30	4.8
		Disagree	584	93.6
Self‐efficacy	Towel sharing	No	457	73.2
		Yes	157	25.2
	Regular health education attendance	No	140	22.4
		Yes	474	76.0
	Confidence in preventing trachoma	No	90	14.4
		Yes	524	84.0

Regarding Perceived Benefits, confidence in specific preventive actions is very high. Mothers overwhelmingly believe in the efficacy of eye drops (98.7%), face washing to prevent the spread of infection (98.4%), and the value of health education in preventing blindness (97.4%). This suggests strong acceptance of core components of the SAFE strategy. The lower perceived benefit of general sanitation practices (59.1%) may indicate a disconnect between understanding specific facial cleanliness and broader environmental hygiene, or a perception of it being less directly controllable. The profile of Perceived Barriers is notably encouraging for program implementation. Deeply entrenched cultural barriers are minimal, with few attributing the disease to divine causes (6.9%) or preferring traditional medicine (9.6%), and social stigma is low (4.8%). The most practical barrier is financial, with 8.0% citing the cost of the SAFE strategy as a concern. This highlights that the primary obstacle is not cultural resistance, but rather economic accessibility, which guides efforts toward subsidizing or providing free materials. Finally, Self‐Efficacy presents a picture of general confidence but also highlights a notable behavioral lapse. While 84.0% feel confident in preventing trachoma and 76.0% attend health education, a substantial 25.2% still report sharing towels. This critical discrepancy between high confidence/attendance and this specific high‐risk behavior points to a gap in translating knowledge into habitual practice. It suggests that education must move beyond raising awareness to actively promote and enable the replacement of specific, entrenched household behaviors. In general, the HBM analysis identifies a clear path for intervention: leverage the high perceived severity and benefits to reinforce behavior change, directly target the knowledge gap on fly transmission, address the financial cost barrier, and design educational programs that specifically aim to transform knowledge into consistent practice, with towel hygiene as a primary behavioral target (Table 2).

### 3.4 Descriptive Statistics for HBM Constructs

The descriptive analysis of the HBM constructs reveals a distinct pattern among participants. There was an overwhelmingly high perception of the severity of trachoma (mean = 0.97, SD = 0.02), coupled with strong beliefs in the benefits of preventive measures (mean = 0.85, SD = 0.06) and relatively low perceived barriers to action (mean = 0.89, SD = 0.05). In contrast, participants reported only moderate levels of perceived personal susceptibility to the disease (*M* = 0.65, SD = 0.20) and self‐efficacy, or confidence in (*M* = 0.97, SD = 0.02), coupled with strong beliefs in the benefits of preventive measures (*M* = 0.85, SD = 0.06) and relatively low perceived barriers to action (*M* = 0.89, SD = 0.05) in their ability to perform preventive behaviors (*M* = 0.68, SD = 0.21). This profile suggests that while the community recognizes trachoma as a serious threat and values preventive actions, a significant portion may not feel personally at risk or confident in their ability to engage in those actions, which could be a critical target for health intervention programs (Table 3).

**Table 3:** Descriptive statistics for health belief model (HBM) constructs (*n* = 624).

HBM construct	Mean	Standard deviation	Min	Max
Perceived susceptibility	0.65	0.20	0.04	1.00
Perceived severity	0.97	0.02	0.91	1.00
Perceived benefits	0.85	0.06	0.66	1.00
Perceived barriers	0.89	0.05	0.75	1.00
Self‐efficacy	0.68	0.21	0.00	1.00

*Note:* All constructs were measured on a scale from 0 to 1, with higher scores indicating a stronger presence of the construction.

### 3.5 The Efficacy of the HBM (Correlation Between Psychological Constructs and Prevention Practice Adherence)

**Figure 2:**
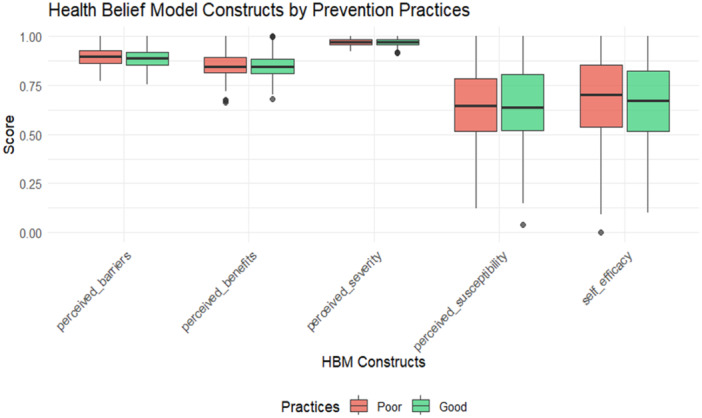
The efficacy of the health belief model: Analyzing the correlation between psychological constructs and prevention practice adherence.

Based on an analysis of prevention practices through the HBM, the data reveal a strong, consistent correlation between the model's core psychological constructs and reported health behaviors, validating its fundamental premise. The Perceived Benefits construct emerges as the most powerful differentiator, suggesting that a strong belief in an action's efficacy is a primary driver for its adoption. Conversely, Perceived Barriers represents the most persistent obstacle, showing the smallest gap between those with “Good” and “Poor” practices, indicating that practical and psychological challenges remain significant even for motivated individuals. The supporting constructs of Perceived Susceptibility, Perceived Severity, and Cues to Action also show strong positive associations. Consequently, this analysis underscores that successful public health interventions must be multifaceted, strategically amplifying the perceived benefits of behavior while actively working to minimize the real and perceived barriers to action (Figure 2).

### 3.6 Correlational Analysis of HBM Constructs and Prevention Behaviors

This correlation matrix reveals a surprising and counterintuitive pattern in the relationships between HBM constructs and prevention behavior. Contrary to the model's theoretical predictions, none of the HBM constructs show a positive correlation with the adoption of prevention practices (prevention binary). All correlations with the prevention binary are negative and exceedingly weak, hovering near zero. The strongest (though still very weak) negative relationship is observed with perceived barriers (*r* = −0.056), followed by self‐efficacy (*r* = −0.034). This suggests that higher levels of perceived obstacles and, paradoxically, higher self‐efficacy, are very slightly associated with a lower likelihood of engaging in prevention behaviors. The constructs of perceived susceptibility, perceived severity, and perceived benefits show virtually no linear relationship with behavior (all *r* < |0.020|).

Inter‐construct Relationships: The correlations between the different HBM constructs themselves are also negligible and near‐zero. There is no evidence of a cohesive structure where, for example, perceiving higher severity is associated with perceiving greater benefits. The strongest relationship among constructs is a weak negative correlation between self‐efficacy and perceived barriers (*r* = −0.074), which aligns with theory (higher self‐efficacy is associated with viewing barriers as less formidable) (Table 4).

**Table 4:** Correlation matrix of health belief model constructs and prevention behavior.

Variable	Perceived susceptibility	Perceived severity	Perceived benefits	Perceived barriers	Self‐efficacy	Prevention binary
Perceived susceptibility	1.000	0.004	−0.017	−0.027	−0.047	−0.003
Perceived severity	0.004	1.000	0.004	−0.022	−0.029	−0.005
Perceived benefits	−0.017	0.004	1.000	−0.030	−0.032	−0.020
Perceived barriers	−0.027	−0.022	−0.030	1.000	−0.074	−0.056
Self‐efficacy	−0.047	−0.029	−0.032	−0.074	1.000	−0.034
Prevention binary	−0.003	−0.005	−0.020	−0.056	−0.034	1.000

*Note:* All values are correlation coefficients (r). Coefficients near zero (|*r*| < 0.1) indicate a very weak or non‐existent linear relationship.

This study presents a detailed descriptive profile of the health beliefs held by mothers in a trachoma‐endemic community, highlighting both strengths and critical gaps. The high levels of perceived severity and perceived benefits are significant assets. Most mothers viewed trachoma as a severe, blinding disease and believed in the efficacy of preventive measures like face washing. This creates a receptive foundation for health messaging, as the community acknowledges the threat and the value of the proposed solution. However, the moderate score for perceived susceptibility suggests a possible “third‐person effect,” where mothers may not personalize the risk. Crucially, the specific knowledge gap regarding flies as a vector (known by only 38.5%) represents a direct loophole in the logical chain of prevention; one cannot avoid a transmission route one does not acknowledge [11].

The correlational findings are particularly instructive. The near‐zero correlations between HBM constructs and prevention practices challenge a simplistic, linear “knowledge‐behavior” model. This indicates that while these beliefs are important, they do not directly or independently translate into action in a straightforward manner. The weak inter‐construct correlations further suggest that these perceptions operate somewhat independently in this context; for instance, believing the disease is severe does not automatically equate to feeling personally susceptible or confident in one's ability to prevent it. This complexity underscores the need for interventions to be multi‐pronged, simultaneously addressing specific knowledge deficits, personalizing risk, and building skills, rather than relying on a single type of persuasive message [12].

The profile of a mother with suboptimal practices, inferred from this analysis, is not necessarily one who is ignorant or dismissive, but perhaps one who knows the disease is serious but does not see her family as highly vulnerable, lacks specific knowledge about environmental transmission, and feels only moderately confident in her ability to maintain hygiene amid daily constraints. Therefore, effective communication must move beyond fear‐based messaging about severity to include skills‐based training (to raise self‐efficacy), localized, visual demonstrations of transmission pathways (to address specific gaps, such as the role of flies), and practical problem‐solving around cited barriers, like water access [12].

## 4 Conclusion and Recommendations

### 4.1 Conclusion

This descriptive and correlational analysis provides a nuanced map of maternal health beliefs related to trachoma in rural Ethiopia. It confirms that while perceived severity and benefits are high, key knowledge gaps and moderate self‐efficacy persist. The absence of strong linear correlations between beliefs and practice highlights the non‐linear, complex nature of health behavior adoption, necessitating the design of sophisticated interventions beyond information dissemination alone.

### 4.2 Recommendations

#### 4.2.1 Develop Targeted Educational Content

Health education materials must explicitly rectify the identified knowledge gap on fly transmission using visual aids (e.g., pictorial stories, flip charts) suitable for low‐literacy populations.

#### 4.2.2 Segment Communication Strategies

Messages should be tailored for mothers with different belief profiles. For those with high severity but low susceptibility perceptions, messaging should focus on personalizing risk (e.g., “Your children playing outside are at risk from flies.”).

#### 4.2.3 Adopt a Multi‐Construct Approach

Design communication campaigns that concurrently address multiple HBM constructs, emphasizing severity, personalizing susceptibility, demonstrating benefits, and modeling actions to build self‐efficacy—rather than focusing on a single factor.

Use Correlational Insights for Prioritization: The independent nature of constructs suggests that interventions can target specific domains (such as boosting self‐efficacy through hands‐on practice) without assuming they will automatically improve perceptions in other areas (like susceptibility).

## Author Contributions

Conceptualization, methodology, writing original draft preparation: Zufan Alamrie Asmare, Chalachew Yenew, Almaw Genet Yeshiwas, Gashaw Melkie Bayeh, Tilahun Degu Tsega, Sintayehu Simie Tsega, Asaye Alamneh Gebeyehu, Getaneh Atikilt Yemata, Zeamanuel Anteneh Yigzaw, Abathun Temesgen, and Abebaw Molla Kebede. Formal analysis, investigation, data curation, visualization (preparation of tables and figures): Habitamu Mekonen, Anley Shiferaw Enawgaw, Berhanu Abebaw Mekonnen, Meron Asmamaw Alemayehu, Getasew Yirdaw, Zekaryas Ewnetu Gashu, and Chalachew Yenew. Approval and Writing – review and editing: All authors.

## Funding

The authors have nothing to report.

## Disclosure

All authors have read and approved the final version of the manuscript. Chalachew Yenew had full access to all the data in this study and takes complete responsibility for the integrity of the data and the accuracy of the data analysis.

## Ethics Statement

Ethical approval for the original survey was obtained from the Institutional Review Board of the University of Gondar (Ref. No. CMHS/5028/2022). Permission was secured from the Andabet District Health Office.

## Consent

Informed verbal consent was obtained from all study participants after explaining the purpose, benefits, risks, and their right to withdraw. Confidentiality was maintained by using anonymous codes and securing data.

## Conflicts of Interest

The authors declare no conflicts of interest.

## Transparency Statement

The lead author, Chalachew Yenew, affirms that this manuscript is an honest, accurate, and transparent account of the study being reported; that no important aspects of the study have been omitted; and that any discrepancies from the study as planned (and, if relevant, registered) have been explained.

## Data Availability

The datasets generated and analyzed during the current study are not publicly available due to containing potentially identifiable participant information. However, they are available from the corresponding author upon reasonable request and with permission from the institutional ethics review board.
